# Yerba Mate—A Long but Current History

**DOI:** 10.3390/nu13113706

**Published:** 2021-10-21

**Authors:** Anna Gawron-Gzella, Justyna Chanaj-Kaczmarek, Judyta Cielecka-Piontek

**Affiliations:** Department of Pharmacognosy, Faculty of Pharmacy, Poznan University of Medical Sciences, 4 Swiecickiego Street, 61-781 Poznan, Poland; aggzella@ump.edu.pl (A.G.-G.); justyna.chanaj-kaczmarek@ump.edu.pl (J.C.-K.)

**Keywords:** Yerba Mate, active compounds, biological activity, functional food, potential toxicity

## Abstract

Bearing in mind the growing interest in Yerba Mate, a comprehensive study has been prepared containing the most important aspects and possibilities of its use. The introduction of the work contains the species characteristics of Yerba Mate, as well as information about the origin and places of cultivation. The next part focuses on the analysis of the composition, pointing to purine alkaloids, polyphenols, saponins, and minerals as groups of active compounds responsible for the clinical activity of Yerba Mate. The review of the results of preclinical and clinical studies indicates activity in relation to the stimulating effect, reducing weight by stimulating lipolysis, cardioprotective, anti-diabetic, and anti-inflammatory effects. The information about the action of Yerba Mate is supplemented by the characteristics of its potential toxicity in terms of PAHs content (in particular benzo[α]pyrene) and preparation as a determinant of increased irritation. The current data on the effects of Yerba Mate and the wide safety margin of its use position this raw material as a valuable component of functional food. The growing frequency of consuming Yerba Mate, conditioned by the availability resulting from the globalization of the market and the information provided about it’s the pro-health effects, will position Yerba Mate’s popularity among wider population groups.

## 1. Introduction

*Ilex paraguariensis* A.St.-Hil., of the Aquifoliaceae family, is a tree whose leaves (*Mate folium*), after being dried and roasted, are used to prepare the Paraguayan tea known as “Yerba Mate”. The genus Ilex comprises about 450 species growing in the tropical regions of South America and Asia. Ilex trees are located exclusively in South America: in northern Argentina, southern Brazil, Uruguay, and Paraguay, where they cover an area of approximately 540,000 km^2^ [1,2,3,4,5]. The holly leaf of *I. paraguariensis* has EMA monographs and, more recently, a pharmacopoeial monograph (in Supplement 9.4 to the European Pharmacopoeia) [6,7,8]. 

*I. paraguariensis* is a subtropical, dioecious, evergreen tree that grows from 8 to 15 m. The hard perennial leaves are 8 cm long, olive green in color, darker on top. They are egg-shaped with a wedge-shaped base, crenate edge, and blunt tip. Paraguayan holly blooms from October to November. It has small, white, silky flowers (usually four-petaled) gathered in inflorescences at the tops of branches. It fruits from March to June. The fruit are red or reddish-brown berries 5–8 mm in diameter, containing 4–5 yellow seeds with a hard shell, which are dispersed by birds. There is a residual embryo in many apparently ripe seeds, so that the period from sowing to germination can be long. The tree requires high humidity throughout the year the rain-fall must not be less than 1200 mm, while is less sensitive to temperature fluctuations and can withstand even −6 °C [1,9,10]. 

The words “Yerba Mate” come from two languages (Spanish and Ketchua), and literally mean “herbs from the calabash”, because mate leaves were brewed in special vessels made of dried calabash fruit. The first to use Yerba Mate in its natural habitat was the Guarani Indians. In the 16th century, the Jesuits came to this area, who not only appreciated the advantages of the drink, but soon (around 1670 years) started to professionally cultivate and trade in the leaves of the Paraguayan holly, hence the drink made from the leaves became known as Paraguayan, missionary, or Jesuit tea. After the dissolution of the order in 1775, the plantations run by the monks were destroyed and became wild. The difficult art of cultivating this plant was resumed only in the middle of the 20th century [1,9,11]. 

The production of Yerba Mate tea in the three main countries (Argentina—the largest producer, Brazil, and Paraguay) is estimated at about 1.4 million tons per year, of which only less than 5% is exported, while the vast majority, as a national product, is destined for domestic consumption, e.g., about 80% in Brazil [3]. 

## 2. Yerba Mate Processing

The cultivation and harvesting of Yerba Mate are carried out in different ways depending on the region. The raw material is obtained from holly trees growing in natural conditions and from plantations or trees planted in the wild. The production process of Yerba Mate tea follows several stages, which, depending on the producer and the desired taste of the tea, differ in duration, temperature, and type of wood used for roasting and drying the product. 

The production process of Yerba Mate tea includes:

- **Raw material harvesting**—fresh (6–12-month-old) leaves and stems are harvested, packaged, and transported to the processing site.
- **Roasting** (blanching)—brief (10 s to 3 min) heating at temperatures up to about 500 °C to inactivate oxidative enzymes (polyphenol oxidase), slow the natural decomposition of plant material, and preserve sensory properties. Traditionally, this process was carried out by direct exposure to an open wood or propane fire in a rotary kiln (cylinder). Today, most furnaces have been replaced by automatic belt dryers that blow hot air into the leaves.
- **Drying**—in order to reduce the moisture content of the leaves to 4.5%, the raw material is placed for 8–24 h in drying chambers where temperatures up to 100 °C and filtered or unfiltered smoke from the burning wood is used.
- **Maturation**—dry product is crushed and placed in cement or cedar chambers for at least 12 months to develop its specific flavor. Maturation significantly increases the concentration of certain components (methylxanthines and polyphenols) and the antioxidant activity of Yerba Mate.
- **Sieving**—separating the twigs from the leaves in order to remix them in specific proportions. The leaves are responsible for the active compounds content and the taste of the drink, while the twigs act as a kind of filter to stop dust from entering the Yerba Mate drinking tubes and reduce the price of the finished product. Different types and brands of Yerba Mate have a specific ratio of leaves and twigs. Yerba Mate Despalada is theoretically without twigs (in reality it contains up to 10% of them), while Yerba Mate Elaborada is a type of dried tea containing about 35% of twigs.
- **Packaging**—the product is crushed before packing and can be flavored with natural fruit essences, such as pomelo juice or other citrus fruits [1,3,10,12,13,14].

The way Yerba Mate is consumed is different in each South American country due to the difference in customs between the native South Americans and the European colonizers of those countries. Traditional Yerba Mate is brewed in vessels called *matero* made of calabash, wood, or porcelain. The dry tea is poured into the *matero* in the amount of 1/4 or a little bit more of its volume, poured with water at the temperature of 65–80 °C to about 2/3 of the vessel’s volume and left for 3–5 min. The drink is taken through a tube called a *bombilla*. The drought can be poured over the water many times, even six or seven times, until it loses its taste. Traditional products consumed in Argentina, Uruguay, and Brazil prepared by pouring hot water over the leaves are referred to as “mate”, “mate “*cocido*”, or “*chimarrao*”. In Paraguay and southwestern Brazil, a popular drink is “*tereré*”, prepared by pouring cold water over the leaves and often served with lemon or other citrus fruit [1,2,3,5,15].

In countries where Yerba Mate occurs in its natural state, the average consumption of dried leaves is estimated at 12–23 g/day (=1 L of drink). The highest per capita consumption of Yerba Mate is noted in Uruguay with 8–10 kg/year, in Argentina about 6–5 kg/year, and in southern Brazil 3–5 kg/year [1,3,9,16]. 

In addition to the traditional Yerba Mate drinks, concentrated extracts are used in the pharmaceutical, cosmetic, and food industries. Increasing interest is attributed to the development of healthier foods, especially those naturally rich in bioactive phenolic compounds with a protective effect against the development of chronic diseases. Based on the Derwent Innovations Index database, the number of patent applications for Yerba Mate-based products has been steadily increasing since 2008 [17], including supplements to reduce glucose or cholesterol levels, treat metabolic syndrome and obesity, as well as against oxidative stress and its consequences. One such patented preparation for controlling weight loss in humans is “YGD” containing 112 mg of Yerba Mate, 95 mg of Guarana (seeds of *Paullinia cupana*), and 36 mg of Damiana (leaves of *Turnera diffusa* var. *aphrodisiaca*) [18]. In addition, mate leaves are used to produce cold non-alcoholic beverages, as ingredients of energy drinks and products promoting alcohol and acetaldehyde degradation. 

Interestingly, a promising role for Yerba Mate has been confirmed by recent studies of biodegradable and functional food packaging. The addition of Yerba Mate extract to hydrolyzed starch membranes, due to its antioxidant activity and color change at different pH, significantly increases plasticization and accelerates the decomposition of packaging [19]. 

## 3. Chemical Compounds

Yerba Mate contains numerous chemical compounds, including nutrients: carbohydrates (80.71%), proteins (4.09%), and fats (0.90%). In addition, it contains numerous secondary metabolites, especially purine alkaloids (caffeine, theobromine), polyphenols (phenolic acids, flavonoids), and terpenes (saponins, carotenoids), as well as minerals and vitamins [1,2]. 

### 3.1. Purine Alkaloids 

Yerba Mate is a rich source of methylxanthines, especially caffeine (1–2% of dry weight), theobromine (0.3–0.9%), and trace amounts of theophylline. These compounds are found mainly in leaves (3.15–3.5 mg/g) and in small concentrations in stems. They are responsible for the characteristic bitter taste and stimulating effect of the drinks. The caffeine content in a cup (about 150 mL) of Yerba Mate tea is comparable to that in a cup of coffee and is about 80 mg [1,11,20]. However, the amount of alkaloids in beverages is variable and depends on the type of raw materials used and how the brew is prepared. In a study of teas from Uruguay, Argentina, and Brazil (three from each country), it was shown that Uruguayan teas had the highest contents of caffeine and theobromine, followed by Argentine teas and the lowest Brazilian. Caffeine content in Yerba Mate can vary from 25 to 175 mg/g and theobromine from 6 to 28 mg/g of dry mass [3,15,21]. The amount of caffeine consumed in 100 mL of the cold-prepared drink (*terere*) was found to be about 2.5 times higher than that of the hot water-brewed *chimarrao* drink. Compared to other products, the amount of caffeine in 100 mL of *terere* and *chimarrao* was the same as in 24 and 10 mL of espresso coffee, 277 and 118 mL of cola, 80 and 34 mL of energy drink, or 1.1 and 0.5 g of guarana powder, respectively, while the amount of theobromine in these two drinks was equivalent to 14.5 or 6.2 g of milk chocolate [22]. A similar relationship was found in subsequent studies, in which caffeine concentration increased with brewing time, obtaining the highest content in beverages poured over water at 70 °C, brewed for 45 min. Brewing with boiling water was very inefficient and, regardless of the time, the drink had two times lower caffeine concentration [16]. Purine alkaloids content depending on procedures used to prepare infusions is presented in Table 1.

### 3.2. Polyphenols 

The leaves of Yerba Mate contain several polyphenolic compounds, mainly phenolic acids, of which chlorogenic acid is the dominant one, occurring most often in the form of three isomeric compounds. In freshly harvested raw materials, the amount of chlorogenic acid varies between 46–81 μg/mg dry mass in leaves and 32–79 μg/mg in stems [23,24]. In aqueous and alcoholic extracts from green and roasted Yerba Mate, the presence of chlorogenic acid (caffeoylquinic acid), caffeic acid, quinic acid, dicaffeoylquinic acid, and feruloylquinic acid was confirmed. In roasted Yerba Mate (as opposed to green one), the presence of caffeoylshikimic and dicaffeoylshikimic acid (previously found in dried plums) was additionally found [23,25]. The total polyphenol content, expressed as chlorogenic acid equivalent, most often ranged from 40 to about 100 mg/g dry mass or, expressed as gallic acid equivalent, from 90 to 176 mg/g dry mass [3,21,23,26,27]. 

In extensive studies, the content of total polyphenols determined in Yerba Mate from Argentina, Brazil, Paraguay, and Uruguay, which was obtained from natural forests or plantations, dried with hot air or wood smoke, was 100–180 mg/g in terms of chlorogenic acid, which means that a cup of tea prepared from a teaspoon (5 g) of Yerba Mate can provide 0.5–0.9 g of polyphenols. Raw materials from sunny plantations in Paraguay were found to be the richest in polyphenolic compounds [1]. In the study by Colpo et al., 15 consecutive brews were prepared from nine Yerba Mate teas (three raw materials each from Uruguay, Argentina, and Brazil). The highest amount of polyphenols was found in the second brews, with drinks from Uruguayan teas containing approx. 3–5 times more polyphenols compared to the poorest Brazilian raw materials. In 100 mL of the richest drinks from Uruguay, Argentina, and Brazil, there were 1451 mg, 967 mg, and 599 mg of polyphenols per gallic acid, respectively [15]. The second extracts were also found to be the richest in polyphenols in assays conducted on 30 consecutive brews of five Brazilian Yerba Mate. Additionally, it was noted that the amount of polyphenols, as well as methylxanthines, was higher in cold-prepared beverages than those brewed with hot water. The eighth water extract of “*terre*” had more polyphenols than the third extract of “*chimarrao*” [22]. The content of polyphenols in infusions prepared with various procedures is presented in Table 1.

In a study by De Mejia et al., the contents of chlorogenic acid and its isomers (neo- and cryptochlorogenic acid) determined in 13 traditional Yerba Mate from Argentina and Paraguay were 43.1, 58.7, and 36.1 μg/mg, respectively. In addition, the presence of 3,4-, 3,5-, and 4,5-dicavolylquinic acid was also found in the samples [21]. The HPLC method developed to determine the content of polyphenolic compounds in different brands of “*chimarrao*” and “*terre*” teas (with different leaf and stem contents) also confirmed the highest content of chlorogenic acids in Yerba Mate. Using this method, the content of nine phenolic acids was determined in the studied beverages: caffeic; chlorogenic; 3,4-, 3,5-, and 4,5- dicavolylquinic; gallic; syringic; p-coumaric; and ferulic acids. Maximum chlorogenic acid content was observed when 2 g of Yerba Mate was poured with 300 mL of 95 °C water and brewed for 16 min [28,29].

The following flavonoid compounds are also found in Yerba Mate: rutin, quercetin 3-rhamnoside and 3-glucoside, kaempferol 3-rhamnoside and 3-glucoside, and luteolin diglycoside. Their total content per catechin is about 70 mg/g d.m., while the amount of rutin alone determined in 23 traditional Argentine and one Paraguayan Yerba Mate was 15–35 μg/g extract [12,21,23,27]. 

**Table 1:** Purine alkaloids and polyphenols content in Yerba Mate infusions.

	Preparation of Yerba Mate Infusions	Repeats	Content (mg/100 mL Infusion)	
			Purine Alkaloids	
			Caffeine	Theobromine
De Morais et al. [20]	50 mg of green or 20 mg of roasted Yerba Mate/1 mL of boiling water, infused for 10 min	1st	15.74	4.81
			10.99	2.70
			respectively	respectively
Meinhart et al. [22]	*chimarrao*: 85 g of Yerba Mates (smooth, traditional, native, course-ground) brewed with 150 mL water at 75 °C for 30 s	1st	13.7–26.4	2.8–6.4
		2nd	6.5–19.0	1.7–4.4
		3rd	2.3–13.6	0.6–3.1
		4th	1.6–11.6	0.4–2.9
		10th	1.0–7.3	0.3–1.6
		18th	1.3–5.7	0.3–1.3
		30th	1.5–6.6	0.3–1.6
	*terere*: 50 g of Yerba Mate extracted with 180 mL of cold water (11 °C) for 30 s	1st	35.8	8.2
		2nd	56.5	12.3
		3rd	48.2	10.4
		4th	33.2	7.0
		10th	12.2	2.4
		18th	6.1	1.1
		30th	2.9	0.5
Kruszewski et al. [16]	3.5 g Yerba Mate brewed with 100 mL water at 70 °C for:	1st		-
	15 min		15.0	
	30 min		17.5	
	45 min		25.5	
	60 min		25.8	
	3.5 g Yerba Mate infused with 100 mL boiling water for:	1st		-
	15 min		11.8	
	30 min		12.3	
	45 min		13.1	
	60 min		15.8	
			**Total polyphenols content**	
Bastos et al. [25]	5 g of green or roasted Yerba Mate were extracted continuously for 4 h in a Soxhlet apparatus with 100 mL of water at 97 °C	1st	733 and 671 *, respectively	
De Morais et al. [20]	50 mg of green or 20 mg of roasted Yerba Mate/1 mL of boiling water, infused for 10 min	1st	551 and 174 **, respectively	
De Mejía et al. [21]	2.7 g of traditional Yerba Mates brewed with 250 mL of boiling water for 10 min	1st	130–260 *	
Meinhart et al. [22]	*chimarrao*: 85 g of Yerba Mates (smooth, traditional, native, course-ground) brewed with 150 mL water at 75 °C for 30 s	1st	120.5–235.6	
		2nd	55.5–121.1	
		3rd	21.9–104.3	
		4th	12.3–62.9	
		10th	11.0–31.4	
		18th	12.8–29.2	
		30th	14.5–38.6 *	
	*terere*: 50 g of Yerba Mate extracted with 180 mL of cold water (11 °C) for 30 s	1st	235.3	
		2nd	350.0	
		3rd	268.4	
		4th	208.5	
		10th	72.3	
		18th	36.3	
		30th	19.7 *	
Colpo et al. [15]	85 g Yerba Mates (Argentinean, Brazilian and Uruguayan brands) brewed with 70 mL water at 85 °C for 1 min	1st	408–1185	
		2nd	220–1451	
		5th	97–1233	
		10th	72–761	
		15th	56–514 *	

* the content expressed as gallic acid equivalent (mg GAE/100 mL infusion), ** the content expressed as chlorogenic acid equivalent (mg CGA/100 mL infusion).

### 3.3. Saponins 

The saponin compounds present in mate leaves belong to bitter, well water-soluble triterpene saponins, derivatives of ursolic acid, which have been named mate saponins 1, 2, 3, 4, and 5 [1,6]. Their total content is 7.0 mg/g in green and 6.60 mg/g in roasted leaves [20]. According to other studies, the content is 11.7 mg/g and the dominant compound is mate saponin 1 (ursolic acid 3-O-gluco-arabino-28-glucoside, 5 mg/g) and mate saponin 2a and 2b (isomeric ursolic acid 3-O-gluco-arabino-28-glucosides, about 3 mg/g each), while mate saponins 3, 4, and 5 are present only in trace amounts. It was also noted that they are not as well soluble in water as in organic solvents, hence their levels in the infusions to which they impart a specific flavor are not as high as in the raw material [11,30,31]. 

Guaiacin B and nudicaucine C, which are analogues of mate saponin 1 and 2, derivatives of another aglycone, oleanolic acid, have also been identified [6,12,32]. The MALDI- and LDI-MS method developed by Petroselli et al. to assess the quality of the raw material now allows the detection of known saponins in *I. paraguariensis* leaves, as well as new ones not previously described as components of commercial Yerba Mate. The method can also reveal the presence of contaminants and/or food additives, such as cyclodextrins α and β [33]. 

### 3.4. Minerals

The primary macronutrients in Argentine Yerba Mate leaves are K, Ca, Mg, and P in amounts of 11.35, 7.69, 6.99, and 1.37 mg/g, respectively, while, among the trace elements, manganese and iron are the most abundant (0.68 and 0.12 mg/g), while aluminum and silicon (0.64 and 0.33 mg/g) among the ultra-trace elements [3,34,35,36]. In a study of 54 teas from Argentina, Brazil, Paraguay, and Uruguay, similar (irrespective of country of origin) contents of Ti, Ni, As, Mo, U, Li, and Be were determined, while the amounts of K, Cr, Ca, Mg, Co, Al, Fe, Rb, Mn, P, Sr, La, Cu, Ce, Ba, Pb, and Zn in the different Yerba Mate teas varied according to the growing conditions of the raw material, soil, weather, and processing practices. In this study, average concentrations of the toxic elements, i.e., cadmium, arsenic, and lead, were also determined below the maximum acceptable levels, except for one Brazilian tea in which the Cd content (0.491 μg/g) exceeded the acceptable standard by almost 20%. No toxic elements, such as Sb, Se, Ag, and Bi, were found in any of the teas tested [35]. The method developed by Marcelo et al. based on the determination of the content of 24 essential minerals as chemotaxonomic components for Yerba Mate is useful for the identification of the country of origin and quality control of Yerba Mate, and also allows the detection of adulteration of the raw material due to the addition of other herbs [37]. 

### 3.5. Other Compounds 

Yerba Mate also contains vitamins, mainly water-soluble ones in 100 g of dried leaves; about 22 mg of vitamin C; and 5.5, 1.8, and 0.7 mg of vitamin B1, B2 and B6, respectively, were determined [3]. According to some authors, the raw material also contains small amounts of vitamins A and E [1,9]. 

Fatty acid analysis of Yerba Mate from Argentina, Brazil, and Paraguay showed significant amounts of linoleic and α-linolenic acids (250 and 600 ug/mL of infusion, respectively). A significantly higher amount of linoleic acid was observed in beverages prepared with water at room temperature compared to infusions with water at 85 °C, while there was no such difference for α-linolenic acid [38]. 

Other studies have identified 23–30 components of Yerba Mate essential oil. It turned out that the process of mate roasting leads to significant changes in the composition of essential oils. The amounts of compounds responsible for the floral aroma are significantly reduced in this process with limonene from 18.2 to 5.4%, and linalool is practically oxidized to linalool oxides. In turn, roasting produces new compounds, such as methylfurfural and furfural responsible for the sweet and smoky flavor and the brown color of the drinks. The main compounds identified in the essential oil of green Yerba Mate were limonene (18.2%), linalool (12.2%), and geranyl acetate (7.1%), while those of roasted mate were geranyl acetate (11.4%), limonene (5.4%), and β-(E)-ionone (4.8%) [26]. 

The most important steps in Yerba Mate production process are the roasting and the final drying, which take place directly over the flame or in the smoke of the burning wood. During these processes, polycyclic aromatic hydrocarbons (PAHs) are formed as a result of the degradation (pyrolysis) of compounds present in the raw material and the burnt wood; these are highly toxic (carcinogenic) compounds, whose content in the final product should be as low as possible. The determined level of 16 PAHs in the samples taken at different stages of Yerba Mate production, from three different locations in southern Brazil, was 0.4–9.0 mg/kg, i.e., the highest during drying. The less toxic 2–4 ring compounds (including phenanthrene, anthracene, pyrene, and fluoranthene) predominated in all samples, while the more toxic 5–6 ring PAHs were present at low concentrations. The determined content of benzo[α]pyrene, one of the most toxic components of smoke, in various Yerba Mate products ranges from 11.9–99.3 µg/kg [14,39,40]. 

## 4. Pharmacological Activity 

The leaves of Yerba Mate have been used for centuries to prepare ordinary beverages for consumption, and it was not until the late 20th century that scientific research began to test its chemical composition and effects on the human body. Nowadays, it has even been suggested that Paraguayan tea is a better alternative to coffee, and the interest in its consumption is mainly concerned with its stimulating effect on the central nervous system induced by the consumption of caffeine. 

The effect of *I. paraguariensis* beverages on the human body is also due to the presence of polyphenols with antioxidant properties and saponins, as confirmed by numerous in vitro, in vivo, and even clinical studies. Yerba Mate, as a powerful antioxidant, lowers cholesterol, prevents peroxidation and lowers lipids in the blood and tissues, so it can be used to reduce obesity, hypertension, and diabetes. It also has anti-inflammatory and antibacterial activity, and even prevents certain types of cancer. Due to its thermogenic properties, the consumption of Yerba Mate has also become very popular among athletes [1,9]. 

### 4.1. Stimulating Effect

The stimulating properties of Yerba Mate have long been known to the indigenous people of South America, who regularly consume this beverage. Yerba Mate contains water-soluble caffeine, which stimulates the cerebral cortex, so when used as a tonic, it relieves mental and physical fatigue, improves memory and concentration, improves reaction time and alertness, and alleviates the negative effects of exposure to stress. In addition to stimulating the CNS, caffeine stimulates the heart and muscles and speeds up metabolism and oxygen uptake by body tissues, so it has a significant effect on various metabolic functions, such as the feeling of satiety, thermogenesis, and fat oxidation. On the other hand, short-term side effects from consuming excessive amounts of caffeine (0.5–1.5 g) are known to include gastrointestinal upset, and are also known to increase heart rate and irregularity, blood pressure, diuresis, psychomotor agitation, anxiety, and insomnia. The lethal dose of caffeine for an adult is 10–12 g. The amount of caffeine found in a cup of Yerba Mate is similar to the amount in a cup of coffee (about 80 mg), but the typical method of consuming this drink of repeatedly pouring extra water into the “mate” can result in an intake of more than 260 mg of caffeine per serving. Regular consumers of Yerba Mate admit that the brew gives energy while not causing nervousness, as sometimes happens after drinking coffee [1,5,6,11,12,16]. 

### 4.2. Antioxidant Capacity

Numerous in vitro and in vivo studies in animal models, as well as clinical trials, show that polyphenol-rich yerba extracts, especially derivatives of caffeoylquinic acid, have strong antioxidant properties that reduce the likelihood of diseases caused by oxidative stress [6,7,9,10,12,20,23,25,41,42]. 

Green and roasted Yerba Mate extracts showed in vitro high antioxidant activity comparable to synthetic antioxidant (BHT). Additionally, the similar activity of both extracts indicates that the roasting step, although it modifies the profile of volatile and phenolic compounds in the brew, does not cause a loss of antioxidant properties [26]. Green and roasted Yerba Mate extracts (tested by DPPH, ORAC, and FRAP) possessed strong antioxidant activity highly correlated with polyphenolic compound content similar to analogous green tea extracts [15,23,25,43]. In contrast, comparable DPPH free radical scavenging values, determined for 13 samples of traditionally brewed Yerba Mate, were similar to the activity of gallic acid at a concentration of 20 mg/mL [21]. Similarly, in another study, where in addition to DPPH radical scavenging, high catalase activity was also found, confirms the antioxidant activity due to polyphenol content [2]. 

The antioxidant potential of Yerba Mate was demonstrated in a study involving fourteen healthy volunteers who ingested orally 3 × 750 mg of spray-dried raw material extract over a period of 60 days. There was an increase in the antioxidant capacity of blood serum by about 16% on the 7th and 30th day of the study and an increase in antioxidant biomarkers (GSH, SOD, CAT) with a simultaneous decrease in lipid peroxidation biomarkers, i.e., lipid hydroperoxides and substances reacting with thiobarbituric acid [44]. 

### 4.3. Effects on Lipid Metabolism 

A very large amount of research, including clinical trials, has focused on the study of lipid peroxidation and, more specifically, the oxidation of LDL in plasma, which plays a key role in the formation of atherosclerosis. 

Ex vivo studies low-density lipoprotein LDL obtained from human plasma before and after drinking Yerba Mate have shown that, due to the presence of antioxidants, which are absorbed into the bloodstream after consuming the drink, there is reduced oxidation of LDL, leading to a reduction in the accumulation of cholesterol in the walls of blood vessels [45]. The prevention of atherosclerosis has also been confirmed in in vitro studies and with healthy volunteers. The levels of paraoxonase-1 (PON-1), an antioxidant enzyme carried by HDL that inhibits the formation of atherosclerotic plaques by preventing oxidation of LDL, were checked. It was proven that extracts of *I. paraguariensis* rich in polyphenols, especially chlorogenic acid, prevented the loss of PON-1 activity. Both in vitro and in subjects consuming Yerba Mate, a strong inhibition of LDL oxidation was observed, which was mainly explained by an increase in PON-1 activity by about 10% compared to subjects consuming coffee with milk instead of Yerba Mate [46]. Animal studies have also confirmed that *I. paraguariensis* leaf extracts inhibit the development of atherosclerosis; in both rabbits fed with a high cholesterol diet and rats on a hypercholesterolemic diet, serum cholesterol and triglyceride levels in these animals were reduced [47,48]. Green or roasted Yerba Mate (both in vitro and in clinical studies) also significantly increases the relative expression of paraoxonase-2 and mRNA in monocytes and macrophages, which may prevent cellular oxidative stress [49]. 

The improvement in serum lipid parameters was also confirmed in a clinical study involving 102 subjects (based on baseline values of serum lipids and lipoproteins divided into three groups: I—normolipidemic; II—with dyslipidemia elevated LDL and triglycerides, who were not receiving treatment; and III—with hypercholesterolemia treated with statins). After 40-day consumption of 1 L/day of Yerba Mate in three doses, a decrease in LDL fraction was observed in all subjects (in group I and II by about 8.5%, and in group III by 13%) and an increase in HDL fraction was observed in group II by 4.4% and in group III by 6.2%. The probable mechanism of LDL lowering by Yerba Mate is the blockade of cholesterol absorption in the small intestine and/or inhibition of cholesterol synthesis in the liver, which may be attributed to the presence of saponins, polyphenolic compounds, and caffeine. The demonstration that drinking Yerba Mate provides additional reduction in LDL cholesterol in hypercholesterolemic subjects who were on stable statin therapy suggests that the beverage may not only reduce the risk of cardiovascular disease but may also be used in anti-atherosclerotic therapy [20]. In another study involving 15 healthy female students from San Paolo who consumed a drink with 5 g of Yerba Mate once a day for 15 days, the plasma susceptibility to oxidation and gene expression of antioxidant enzymes were checked. During the experiment, strongly reduced lipid peroxidation and significantly increased plasma antioxidant levels and higher expression of antioxidant enzyme genes (SOD, CAT and GPx) were observed, especially after prolonged consumption of the beverage. It has been observed that regular consumption of Yerba Mate can increase the antioxidant protection of plasma and blood in patients with atherosclerosis by regulating mechanisms to combat oxidative stress [41,50]. 

Pereira et al. demonstrated in vivo that Yerba Mate also modulates perimenopausal oxidative stress. Erythrocytes and liver cells isolated from female perimenopausal rats treated with Yerba Mate had unaltered estrogen levels, but markedly increased gene expression of the antioxidant enzymes SOD and GPx and reduced MDA, while CAT expression was elevated only in liver cells [51]. A preliminary case control study of 95 postmenopausal women who consumed more than 1 L of mate infusion per day showed significantly fewer diagnoses of coronary heart disease, dyslipidemia, and hypertension, as well as lower serum glucose levels [52]. The effects of Yerba Mate on bone health were tested in a program to prevent and treat osteoporosis. The bone health of 146 postmenopausal women who drank at least 1 L of Yerba Mate tea daily for at least 4 years was compared with the bone health of the same number of women (same age and time after menopause) who did not drink Yerba Mate. The Yerba Mate drinkers had 9.7% higher bone mineral density (BMD) of the lumbar spine and 6.2% higher BMD of the femoral neck, suggesting a protective effect of chronic Yerba Mate consumption on bone packing [53]. 

In a study by Barg et al., the effects of Yerba Mate on damage induced in rats ex-posed to UV radiation were examined [54]. After 7-day UV exposure, DNA damage in peripheral blood and oxidative disorders (increased lipid peroxidation) in rat skin were observed. Administration of Yerba Mate for drinking and as a gel on the skin during UV exposure prevented this damage. Previous studies have also shown a lack of genotoxicity of Yerba Mate to liver, kidney, and bladder cells, and an increase in DNA resistance in liver cells to DNA strand breaks caused by H2O2. Hence, regular consumption of Yerba Mate with antioxidant and anti-genotoxic properties may protect against damage and facilitate DNA repair [9,42,54].

### 4.4. Cardioprotective Effects 

Consumption of Yerba Mate may affect heart function. After consumption of Yerba Mate tea, antioxidant compounds are absorbed and appear in the circulating plasma where they exert antioxidant effects [55]. By inhibiting lipid peroxidation in individuals with elevated LDL levels, these compounds slow the progression of atherosclerosis and promote vascular relaxation. Long-term consumption of Yerba Mate tea independent of dietary intervention increases plasma antioxidant protection in dyslipidemic patients [41,48,50,55]. The polyphenol-rich Yerba Mate drink prevents the loss of anti-atherosclerotic function of HDL, and this results in an increase in the activity of the antioxidant enzyme with cardioprotective effects, known as paraoxonase-1 [46]. The cardioprotective effects of *I. paraguariensis* are associated with high concentrations of polyphenols that reduce reductive stress responsible for elevated lipid peroxidation and protein carbonyls, and inhibit exercise-induced cardioprotection. The reductive stress induced by Yerba Mate in heart tissue leads to induction of cardioprotection [56]. In a clinical study of 23 healthy subjects who consumed 500 mL of cold (~3 °C) or hot Yerba Mate tea (~55 °C), it was found that consuming a beverage at a low temperature induced better cardioprotective effects on cardiovascular function, did not increase cardiac work, and even decreased myocardial oxygen demand compared to the same subjects drinking hot Yerba Mate [57]. 

### 4.5. Effects of Weight Reduction 

Methylxanthines are responsible for the thermogenic properties of Yerba Mate, which, by accelerating metabolism, increase fat oxidation and energy expenditure. During various sports exercises, fat metabolism increases several times; hence, Yerba Mate drinks are recommended for athletes and physically active people who want to lose weight [58]. Combining Yerba Mate intake with prolonged exercise for “fat loss” increases fatty acid oxidation and improves satiety and mood compared to exercise alone, as observed in an experiment involving 12 healthy, active women (30-min exercise) consuming 2 g of Yerba Mate every day [59]. 

In the treatment of obesity, it is important to reduce the absorption and storage of lipids by, among others, inhibiting pancreatic lipase. The results of in vitro studies showed a strong inhibition of human as well as animal pancreatic lipase activity by Yerba Mate infusions, while inhibition of body weight gain and reduced serum triglyceride and LDL cholesterol levels, as well as lower hepatic lipid content, were observed in in vivo study after a 16-week treatment of obese mice with Yerba Mate tea. The results of another in vivo study showed that antioxidants present in Yerba Mate tea administered to mice protect unsaturated fatty acids present in the liver from oxidation [9,60]. 

The effect of Yerba Mate extract on weight loss was confirmed in vivo study in mice in which obesity was previously induced with a high-fat diet. In the study, it was observed that Yerba Mate has the ability to reduce preadipocyte differentiation and lipid accumulation in adipocytes, which decreases the rate of adipose tissue growth, reduces weight gain, and thus nullifies the risk of obesity. It also reduces serum and liver cholesterol and triglyceride levels, as well as blood glucose levels. It also reduces appetite, while it increases basal metabolism, resulting in faster fat burning [61,62]. Yerba Mate inhibits triglyceride accumulation and modulates the expression of genes regulating adipogenesis that are altered in the obese state and restores their normal expression levels. Results obtained in vitro and verified in vivo in mice with obesity induced by a high-fat diet showed that Yerba Mate extract down-regulates the expression of CREB-1 and C/EBPα, while up-regulates the expression of genes associated with inhibition of adipogenesis, including DLK1, GATA2, GATA3, KLF2, LRP5, PPARγ2, SFRP1, TCF7L2, Wnt10b, and Wnt3a [41,63,64,65]. 

A review by Gambero and Ribeiro collected numerous publications describing hu-man or animal studies on the potential use of Yerba Mate in the fight against obesity. Already the first cited clinical study from 2001 proved that Yerba Mate significantly prolongs gastric emptying time, influencing the feeling of satiety, and resulting in a significant weight loss during the 45 days of the experiment in overweight patients. The subsequent tabulated studies show that the use of Yerba Mate can be useful in the fight against obesity by improving lipid parameters in humans and animals [65]. The synergistic effect of the “YGD” preparation and inulin-based soluble fermentation fiber was also observed, consisting of slowing down the stomach emptying and inducing a feeling of satiety. The study was carried out in normally or slightly overweight women who consumed the preparations 15 min before the two main meals each day (breakfast and dinner) [66]. Visceral fat is also reduced, especially in the liver. Less fattening of internal organs results in their better functioning and reduces the risk of metabolic diseases. It regulates leptin signaling in the hypothalamus and its secretion in adipocytes, which consequently reduces appetite and stimulates the sympathetic nervous system. By normalizing the antioxidant activity of liver enzymes and pancreatic lipase, Yerba Mate reduces lipid peroxidation and absorption [65]. A study by Zapata et al. confirmed that administration of Yerba Mate extract or caffeine derived from it to rats fed a high-fat diet significantly reduces hepatic fat accumulation (22%) and attenuates body weight gain (16%) in these animals [67]. Consuming Yerba Mate at a low temperature (~3 °C) induces greater stimulation of thermogenesis and fat oxidation compared to drinking a hot beverage (~55 °C), while not increasing heart rate, which is particularly important for obese individuals with hypertension and other cardiovascular complications [57]. *I. paraguariensis* also plays an important role in the treatment of obesity by acting on inflammation. After a 30-day Yerba Mate treatment in adult rats, inhibition of excessive appetite, overweight, visceral obesity, and central leptin resistance was observed with concomitant improvement of inflammatory markers in hypothalamus and adipocytes [68]. 

In a clinical study involving 25 obese subjects (BMI < 35), after 12 weeks of Yerba Mate supplementation (3150 mg/day, of which the chlorogenic acid content was 35 mg/g extract), significant decreases in body fat mass and body fat percentage were observed compared to the placebo group, as well as a slight decrease in free fatty acids [62]. Chlorogenic acid, one of the main components of Yerba Mate, which inhibits adipogenesis by reducing the expression of genes that regulate adipogenesis in cells, is responsible for this effect [64]. Another clinical study involved 142 men and women with overweight or obesity, untreated dyslipidemia, and no coronary artery disease who were given 1 l/day of Yerba Mate, green tea, or apple tea for 8 weeks. The Yerba Mate consumption group had a significant increase in serum PON-1 levels (9.7%), an increase in HLD, and a decrease in leptin levels associated with a reduction in BMI, whereas green tea consumption had no effect on serum leptin or PON-1 levels [69]. 

### 4.6. Anti-Diabetic Properties and Protection against Diabetic Complications 

Yerba Mate beverages can be used in the prevention and treatment of diabetes as they combine antioxidant and antiglycation effects against formation of advanced glycation end products (AGEs) [42,70]. In an in vivo study on mice, a decrease in blood glucose, dependent on the amount of Yerba Mate tea administered, was confirmed over a 4-week follow-up [61]. Another study demonstrated that the extract of yerba mate (1.0 g/kg for 8 weeks) inhibited inflammatory markers through the NF- κB pathway. It also restored hepatic insulin signaling in mice with high fat diet-induced obesity [71]. 

A pathogenic factor in microvascular complications of diabetes, arteriosclerosis, damage to skin support fibers (collagen and elastin), or neurodegenerative diseases is the formation and accumulation of advanced glycation end-products (AGEs), i.e., the formation of connections between proteins and reducing sugars, e.g., glucose, leading to changes in protein structure and impairment of its biological function [72]. Inhibition of AGE formation by aqueous extracts of Yerba Mate (1:100 dilution) in vitro was comparable to the activity of standard concentrations of the well-known anti-diabetic agent aminoguanidine, which was not exhibited by green tea [70]. Such a strong antiglycation effect of Yerba Mate is mainly due to polyphenols: chlorogenic acid and caffeic acid, and, to the least extent, saponoside compound known as oleanolic acid. For this reason, Yerba Mate may be a natural herbal adjunct to diabetes treatment as it combines antioxidant and anti-AGE activities [73]. Moreover, it significantly alleviates metabolic syndrome by increasing insulin sensitivity and regulating serum glucose levels and modulating lipid metabolism [65,74]. After a 60-day study, 11 volunteers with type 2 diabetes and 11 prediabetics who consumed 1 L/day of Yerba Mate tea showed significant increases in reduced glutathione and reductions in serum lipid hydroperoxides, and reductions in AGEs were experienced among diabetic patients too [75]. According to the cited studies, Yerba Mate tea consumption attenuates oxidative stress in patients with type 2 diabetes, which may prevent its complications.

On the other hand, in a randomized clinical trial, 148 patients orally administered *I. paraguariensis* leaves (1000 mg) 1× daily in combination with *Morus alba* (50 mg) and chromium picolinate (100 μg). After a period of 3 months, improved glycemic status, in particular for reduced fasting plasma glucose, postprandial glucose, and glycated hemoglobin, was observed, as well as a positive effect on the lipid profile as a result of lowering total content cholesterol, low-density lipoprotein cholesterol, and triglyceride [76]. 

### 4.7. Anti-Inflammatory and Anti-Cancer Effects 

Animal intervention studies have provided strong evidence for the anti-inflammatory effects of Yerba Mate, showing that intraperitoneal or oral administration of mate extracts to mice exposed to tobacco smoke significantly reduced acute lung inflammation [9]. Administration of Yerba Mate to mice chronically exposed to tobacco smoke toxicity reversed lung lesions (fibrosis, alveolar enlargement and hemorrhage) and reduced oxidative damage in tissues [77]. 

In another study, Yerba Mate extract administered to mice was observed to reduce the levels of inflammatory markers, including TNF-α, IL-6, and iNOS in liver and muscle by modulating the NF-kB pathway [71]. The mate saponins present in *I. paraguariensis* leaves are responsible for the anti-inflammatory properties resulting from the modulation of the NF-kB pathway. In vitro (ex vivo) studies have shown that ursolic acid, a component of mate saponins, strongly prevents inflammation, has an apoptotic effect, and inhibits the proliferation of colon cancer cells (HT-29) [30]. On the other hand, studies conducted on an animal model showed chemopreventive effects of both pure mate saponin fraction and Yerba Mate tea in chemically induced colitis in rats. Such effects were due to the ability of Yerba Mate and saponins to inhibit the expression of inflammatory markers iNOS and COX-2 regulated by the NF-kB pathway signaling [31]. Increased levels of the anti-inflammatory cytokine IL-10 and decreased levels of pro-inflammatory cytokines (TNF-α and IL-1β) in adipose tissue were also observed in a rat study, following a 30-day treatment with Yerba Mate [68]. 

The high calorie and excess saturated fatty acids present in a high-fat diet can lead to inflammation. It was found that administration of Yerba Mate extract to rats reversed the pro-inflammatory effects of diet-induced obesity, and the anti-inflammatory effects were due to a decrease in IKK enzyme phosphorylation and NF-κB p65 expression, and an increase in IκBα protein levels and AdipoR1 and IRS-2 expression. In addition, an increase in the IL-10/TNFα ratio was noted in retroperitoneal adipose tissue, liver, and muscle, which also demonstrates the anti-inflammatory effects of Yerba Mate. This study suggests that treatment of diet-induced obesity with Yerba Mate extract reduces both central and peripheral inflammatory processes [78]. 

The study by Correa et al., in which rats with arthritis were given 400 and 800 mg/kg of *I. paraguariensis* extracts for 3 weeks, shows that the applied treatment reduced the levels of free radicals as well as the oxidative damage to proteins and lipids in the liver and brain; increased the plasma antioxidant capacity, glutathione levels, and the ratio of reduced to oxidized glutathione in both liver and brain; as well as reversed the modified activity of xanthine oxidase, superoxide dismutase, and catalase. The beneficial effects of the applied treatment should be explained by the anti-inflammatory and antioxidant effects of Yerba Mate components (e.g., derivatives of chlorogenic acid). Hence, the conclusion that daily consumption of traditional Yerba Mate drinks may be effective in alleviating the symptoms of inflammatory diseases, especially in elderly people [79]. 

Further studies confirm that the anti-inflammatory activity of *I. paraguariensis* is determined by the presence of phenolic compounds, especially rutin and phenolic acids. A polyphenol-rich extract of Yerba Mate (176.1 mg GAE/g) showed in vitro anti-cancer activity similar to pure gallic acid (20 mg/mL). This extract acted as a growth inhibitor of human colorectal adenocarcinoma cells (HT-29 and CaCo-2) [21]. Anti-inflammatory effects, induction of apoptosis, and inhibition of cancer cell proliferation are widely recognized as potential mechanisms for effective treatment of colorectal cancer; therefore, the inclusion of Yerba Mate tea in dietary therapy and colorectal cancer prevention is warranted, especially because of the frequent damage to the gastrointestinal mucosa and kidneys when other chemopreventive agents are used [21,30,31]. In colorectal cancer in mice, oral administration of Yerba Mate extract at a dose of 1.6 g/kg/day significantly inhibited angiogenesis and cancer growth without affecting biological parameters or body weight. The study demonstrated that Yerba Mate extract inhibits cancer cell proliferation, and one mechanism of this action is the induction of apoptosis [4]. 

### 4.8. Other Effects 

Aqueous extracts of Yerba Mate have antifungal effects due to the presence of caffeic acid derivatives, methylxanthines, and rutin. They are also effective antibacterial agents against numerous Gram-negative and Gram-positive bacteria, including *Bacillus subtilis*, *Brevibacterium ammoniagenes*, *Propionibacterium acnes*, *Staphylococcus aureus*, *Streptococcus mutant*, as well as fungi *Saccharomyces cerevisiae*, *Candida utilis*, *Pityrosporum ovale*, *Penicillium chrysogenum*, and *Trichophyton mentagrophytes*. It is probably due to the mixture of numerous compounds, including polyphenols, methylxanthines, and terpenes (linalool, β-ionone, α-terpineol, geraniol, eugenol), which are present in *I. paraguariensis* extracts that cause the antimicrobial activity [11]. Topical application of *I. paraguariensis* extract may provide an alternative to synthetic antifungal drugs for skin damage by the saprophytic fungus Malassezia furfur [9]. It was also confirmed that the triterpenes, i.e., ursolic acid and 4,3-O-[a-D-glucopyranosyl-(1-2)-α-D-galactopyranosyl] oleanolic acid present in the raw material, are toxic to *Trypanosoma brucei* and *T. cruzi* which cause parasitic diseases in humans and animals [1]. Studies on herpes virus cell lines confirmed the inhibition of the development of HSV-1 and HSV-2 by the fraction from *I. paraguariensis* rich in triterpenoid saponins, caffeic and chlorogenic acid, and rutin. The synergistic antiviral activity of these compounds is noticeable by reducing viral infectivity, inhibiting virus entry into cells and virus spreading from cell to cell, and lowering the HSV-1 proteins synthesis [80]. 

Saponins present in dry hydroalcoholic extract and aqueous infusions (1:20) of *I. paraguariensis* were tested for nephrotoxicity, not only do not cause damage, but have a protective effect on kidney status in animals fed with a normal diet and a high cholesterol diet [81]. 

Studies on mice show that oral or intraperitoneal administration of an aqueous extract of *I. paraguariensis* has analgesic and protective effects on the liver (reduces ALT levels) and is therapeutically safe [82]. 

In contrast, a study by Bernardi et al. showed that Yerba Mate extracts prevented dopaminergic death by exhibiting a strong dose-dependent neuroprotective effect on primary midbrain cultures, which may be important for preventing progression of Parkinson’s disease. Chlorogenic acid and theobromine tested individually also had neuroprotective effects, but slightly weaker than Yerba Mate extract as a whole, but stronger than known neuroprotective compounds, such as caffeine [83].

## 5. Yerba Mate as a Functional Food Ingredient

The regular consumption of Yerba Mate beverages is categorized as functional food intake due to its antioxidant, anti-inflammatory, cardioprotective, dyslipidemia-preventing, and insulin-resistance properties. A number of papers have reported results from preclinical and clinical studies, suggesting that Yerba Mate consumption may be an interesting food source for humans to minimize some cardiovascular risk factors [3]. 

Combining physical activity with functional foods enhances metabolic and cardiovascular protective benefits and provides a basis for the prevention of life-style related type 2 diabetes. In a study with different groups of men and women, positive metabolic, satiety, and mood state (focus, energy, and concentration) effects were found at different exercise intensities and after consumption of 1–2 g of Yerba Mate, as well as increased fatty acid oxidation and energy expenditure from fatty acid oxidation by approximately 23%. The combined effects of exercise and mate consumption on metabolic, psychomotor, and appetite control outcomes are essential to designing an optimized lifestyle for obese individuals and those with type 2 diabetes [59,84]. 

As a rich natural source of chlorogenic acid, among others, Yerba Mate can be added to food products to increase their nutraceutical value. Gerke et al. determined the best extraction conditions of the raw material to ensure maximum yield and quality of the aqueous extract (30 min, 80 °C, stirring 400 rpm). The beverages obtained under these conditions are characterized by high concentrations of chlorogenic acid, caffeine, and theobromine, at 591.81, 814.90, and 122.56 mg/L, respectively [85]. Similarly, the study by Fenoglio et al. demonstrated that spray drying of Yerba Mate extract provided lower moisture content, as well as the highest polyphenol content (135.4 mg GAE/g extract) and oxidative stability compared to freeze-dried samples. It was noted that spray-dried Yerba Mate powder can be used in the food industry as an antioxidant food additive for example as it increased the oxidative stability of mayonnaise [86]. 

## 6. Potential Toxicity of Yerba Mate

Apart from the health-promoting effects of Yerba Mate, there are also reports on its toxicity. In studies carried out in countries with a much higher than average consumption of Yerba Mate, the drinking of this beverage has been statistically linked to an increased incidence of cancer, mainly of the esophagus, but also of the oral cavity, pharynx, and larynx, and even of the lungs, stomach, colon, rectum, kidneys, and bladder. The traditional way of making the drink involves pouring water over the same leaves several times and drinking all the resulting portions of the drink. Studies show that only such consumption increases the risk of disease due to the ingestion of toxic polycyclic aromatic hydrocarbons (PAHs), while the occasional cup of Yerba Mate made from a single serving of leaves does not provide any more PAHs than the usual diet [9,14,39,55,87,88,89]. 

It has been shown that PAHs can accumulate in Yerba Mate as a result of soil and atmosphere contamination, as well as a result of their release to the kiln from burnt firewood during raw material roasting at very high temperatures [39]. Numerous studies have shown significant differences in the total PAHs content ranging from 194 ng/g to 9001 ng/g of dried Yerba Mate leaves. On the other hand, the concentration of benzo[α]pyrene is considered to be the main human carcinogen ranging from 0–603 ng/g of dry material (known permissible levels of this compound in food are 0.001–0.01 mg/kg fresh weight), while for water extracts from of Yerba Mate were extracted at most 10% of this compound [90]. The different content of benzo[α]pyrene in Yerba Mate infusions may result from differences in the PAHs content in the dried raw material and in the procedures used to prepare infusions, such as the ratio of dry leaves to water, water temperature, and/or infusion duration [91]. 

It cannot be excluded that drinking hot Yerba Mate infusions causes thermal damage to the esophageal mucosa or acceleration of metabolic reactions, including the formation of carcinogenic substances, which increases the risk of esophageal cancer. There are reports from different regions of the world, including Iran, of an increased incidence of esophageal cancer with consumption of various very hot beverages (coffee, tea) and foods [14,88,92]. A case control study of 131 individuals with esophageal cancer and 381 healthy subjects who declared a history of consuming cold, warm, hot, or very hot Yerba Mate beverages was conducted at a hospital in Paraguay. In the study population, it was observed that it was the temperature of the drink that was an important risk factor for esophageal cancer, rather than the amount or duration of consumption, while drinking the cold drink did not increase this risk. A combined case control study from Argentina, Brazil, Paraguay, and Uruguay (1986–1992 and 1988–2005) involving 1400 cases of esophageal squamous cell carcinoma shows that the incidence of this cancer not only increases with cumulative intake but also with the increase in temperature of the Yerba Mate beverage drunk [93]. The cited conclusions are complemented by a multisite clinical study conducted in Uruguay between 1990 and 2004, which included 13,201 participants from the four largest hospitals in Montevideo. The aim was to examine the association of Yerba Mate consumption with the risk of 13 types of cancer. The study found that drinking hot Yerba Mate beverages was strongly associated with esophageal, lung, and bladder cancers, and significantly with cervical, prostate, and kidney cancers. Cancers of the upper respiratory tract (mouth, pharynx, and larynx) were slightly associated with Yerba Mate consumption, whereas no such effect was observed for stomach, colon, rectum, and breast cancers [94]. 

The results of the cited studies support the hypothesis that the carcinogenicity of Yerba Mate is related to its PAHs content, especially in beverages prepared hot. Changing the manufacturing process of Yerba Mate may reduce this potential risk [95]. 

## 7. Summary

*Ilex paraguariensis* leaves, also known as Yerba Mate, are the main raw material used for the production of traditional South American drinks-dried and roasted Yerba Mate, which have been consumed hot or cold for centuries. 

The effects of Yerba Mate infusions and extracts on human health are directly related to the presence of major chemical compounds, such as polyphenols, methylxanthines, and saponins. Some toxic compounds released from the burning wood during the high temperature roasting of the raw material are also known to limit the consumption of excessive amounts of drinks, especially those prepared hot. 

The studies cited in this paper have provided information on the potential health benefits of Yerba Mate consumption. These include antioxidant (in vitro and in vivo), hypocholesterolemic, weight loss, cardioprotective, anti-diabetic (in type 2 diabetes), anti-inflammatory, and anti-cancer effects. Drinking Yerba Mate beverages seems to be justified, especially as a component of functional foods for chronic diseases with lipid metabolism disorders, and has elevated blood glucose levels, obesity, and even inflammation.
